# Contact activation products are new potential biomarkers to evaluate the risk of thrombotic events in systemic lupus erythematosus

**DOI:** 10.1186/ar4399

**Published:** 2013-12-04

**Authors:** Jennie Bäck, Christian Lood, Anders A Bengtsson, Kristina Nilsson Ekdahl, Bo Nilsson

**Affiliations:** 1 Department of Immunology, Genetics and Pathology, Rudbeck Laboratory C5:3, Uppsala University, Dag Hammarskjölds väg 20, SE-751 85 Uppsala, Sweden; 2 Department of Clinical Sciences Lund, Section of Rheumatology, Skåne University Hospital and Lund University, SE-221 85 Lund, Sweden; 3 Linnæus Center for Biomaterials Chemistry, Linnæus University, SE-391 82 Kalmar, Sweden

## Abstract

**Introduction:**

Patients with systemic lupus erythematosus (SLE) have persistent platelet activation and an increased risk of thrombotic events, which cannot be accounted for by traditional cardiovascular risk factors. Factor (F)XII has a potentially important role in thrombus formation and is triggered by activated platelets. We therefore asked whether the contact system is involved in inflammation and vascular disease (VD) in SLE.

**Methods:**

Fibrin clots were incubated with purified FXII or whole blood, and the activation and regulation of FXII were studied. Plasma from SLE patients with (n = 31) or without (n = 38) previous VD and from matched healthy controls (n = 68) were analyzed for the presence of complexes formed between contact system enzymes and antithrombin (AT) or C1 inhibitor (C1INH) and evaluated with regard to clinical data and laboratory parameters.

**Results:**

Fibrin clots elicited FXII activation and acted as co-factors for AT. In clotting plasma, the levels of FXIIa-AT increased, and FXIIa-C1INH decreased. A similar reciprocal relationship existed in SLE patients. FXIIa-AT was elevated in the SLE patients with a history of VD, while the corresponding levels of factor FXIIa-C1INH were significantly decreased. FXIIa-AT correlated strongly with platelet parameters. The odds ratio for VD among the SLE patients was 8.9 if they had low levels of FXIIa-C1INH, 6.1 for those with high levels of FXIIa-AT, and increased to 23.4 for those with both decreased levels of FXIIa-C1INH and increased levels of FXIIa-AT.

**Conclusions:**

Activation of FXII is elicited by fibrin during thrombotic reactions *in vitro* and *in vivo*, and fibrin acts as a heparin-like co-factor and regulates AT. Patients with SLE had altered levels of FXIIa-serpin complexes, supporting that the contact system is involved in this disease. FXIIa-serpin complexes are strongly associated with previous VD in SLE patients, suggesting that these complexes are potential biomarkers for monitoring and assessing the risk of thrombotic events in SLE.

## Introduction

Patients with the autoimmune disease systemic lupus erythematosus (SLE) have a drastically increased risk of thrombotic events, which are a major cause of morbidity and mortality in these patients. Despite the improved overall survival in patients with SLE over the last few decades, the risk of death in vascular disease (VD) has not decreased [1]. Traditional cardiovascular risk factors can only partly explain this increased risk of VD in SLE [2], and it is likely that inflammatory mechanisms are also involved. The fact that the pathogenic mechanisms involved are not fully understood has contributed to a lack of reliable laboratory parameters for evaluating the risk of thrombotic events.

The contact system, which consists of factor (F) XII, FXI, prekallikrein, and high molecular weight kininogen (HK), is a plasma protease cascade that, when activated, initiates the intrinsic pathway of coagulation (Additional file 1: Figure S1). It has been considered to be of lesser importance for hemostasis, since a deficiency in FXII does not cause any bleeding disorders [3]. Instead, a pathophysiological role for FXII in thrombosis was proposed after FXII-deficient mice were shown to be defective in thrombus formation in arterial beds [4]. FXII and HK have also been shown to bind to and initiate contact activation on endothelial cells via the C1q receptor for globular heads, the urokinase plasminogen activator receptor, and cytokeratin 1 [5-7].

Recently, we demonstrated that activated human platelets trigger FXII-mediated contact activation [8]. These results, combined with the observation that patients with SLE have persistent platelet activation [9-11], led us to hypothesize that the contact system is involved in inflammation and thrombotic conditions associated with SLE. Kallikrein genes have been shown to be associated with glomerular basement membrane-specific, antibody-induced nephritis in mice and with lupus nephritis in humans [12,13], supporting the possible involvement of the contact system. A reduction in FXII plasma levels occurs in SLE patients, concomitant with the appearance of anti-FXII antibodies [14,15], further corroborating a role for the contact system in SLE.

The proteases of the contact system circulate in the blood as zymogens, and after contact activation have been initiated and enzyme activity has occurred, the enzymes quickly form complexes with their inhibitors, making it very difficult to measure free contact enzymes in plasma (Additional file 1: Figure S1). Antithrombin (AT) and C1 inhibitor (C1INH) are members of the serpin protein family [16]. AT is the main inhibitor of the coagulation cascade and inhibits thrombin, FXa, FIXa, et cetera. Its activity is regulated by the glycosaminoglycan (GAG) heparin, which contains a pentasaccharide with a specific sequence of sulfated monosaccharides that upon binding to AT augments the antithrombin activity up to 300-fold. Heparin is present in mast cells and basophilic granulocytes and in the form of heparan sulfate on endothelial cells, but heparin is not available in blood or during blood clotting. In contrast to previous reports stating that FXIIa is mainly inhibited by C1INH, we have shown that the FXII activation triggered by activated platelets and during clot formation is regulated by AT. Only FXII activated by other surfaces (such as artificial material surfaces) is regulated by C1INH [17], which suggests that there is a physiological heparin-like mechanism by which AT is regulated during blood clotting.

In the present study, we investigated the contact system activation in SLE patients by quantifying complexes formed between contact activation enzymes and the serpins AT and C1INH in blood plasma. These complexes were evaluated with regard to their potential correlation with clinical data and with markers of platelet, coagulation, and complement activation. Our primary objective was to determine whether the contact system is activated in SLE patients and, based on our previous studies, to determine whether FXIIa-AT complexes might serve as a new potential biomarker for platelet activation and VD in SLE.

## Methods

### Patients

During routine clinical visits to the Department of Rheumatology at Skåne University Hospital (Lund, Sweden), 69 SLE patients were recruited for blood sampling and laboratory analyses. All patients fulfilled at least four American College of Rheumatology (ACR) classification criteria for SLE [18]. Patients with a known history of VD were preferentially selected, such that 45% of the 69 SLE patients had a history of VD, defined in this study as one or more previous episodes of either myocardial infarction, arterial thrombosis (12/13 with cerebrovascular incidents), or venous thrombosis (pulmonary embolism or deep venous thrombosis), as defined by the Systemic Lupus International Collaborative Clinics/American College of Rheumatology Damage Index [19]. The median time between blood sampling and the last VD event was 8 years. An overview of the clinical characteristics at the time of blood sampling is presented in Additional file 2: Table S1 [20]. Healthy age- and sex-matched volunteers with no history of VD served as controls (n = 68). All of the patients and the controls were Caucasians, except for two individuals of Asian origin among the controls and one individual of Asian and two of Hispanic origin among the SLE patients. Autoantibodies and complement levels in serum were measured according to routine analytic protocols used at the Department of Clinical Immunology, Skåne University Hospital, Lund, Sweden.

### Ethical permission

The study was approved by the regional ethics board at Lund University (LU 378–02). All patients have given written consent to the study.

### Preparation of blood

Fresh human blood, obtained from healthy volunteers who had received no medication for at least 10 days, was collected in vacutainer tubes containing either citrate, ethylenediaminetetraacetic acid (EDTA) (BD Biosciences, San Jose, CA, USA) or lepirudin (added to a final concentration of 50 μg/mL; Schering AG, Berlin, Germany). Blood was centrifuged at 150 × *g* for 15 minutes at room temperature (RT) to produce platelet-rich plasma (PRP) and twice at 2200 × *g* for 15 min to produce platelet-poor plasma (PPP). The plasma was stored at -70°C.

### Detection of P-selectin, platelet intracellular proteins, and platelet-leukocyte complexes

P-selectin (CD62P) exposed on the surface of platelets, IFN-induced transmembrane protein 1 (IFITM1) and protein kinase, IFN-inducible double stranded RNA dependent activator (PRKRA), and platelet-leukocyte complexes were detected by flow cytometry as previously described by Lood *et al*. [20].

### ELISA

The following parameters were measured in EDTA plasma as previously described: FXIIa-C1INH, FXIa-C1INH, FXIIa-AT, FXIa-AT [21], thrombospondin-1 (TSP-1), and thrombin-antithrombin (TAT) [17].

### Plasma protein concentration

C1q, C3 and C4 were measured by nephelometry (Immage, Beckman-Coulter Inc, Hialeach, FL, USA).

### Activation of FXII by fibrin clots

Fifty mIU of thrombin (Hoffmann-La Roche, Basel, Switzerland) was incubated with 0.09, 0.4, or 0.7 μg of fibrinogen (Haemochrom Diagnostica, Mölndal, Sweden) for 10 minutes at 37°C at the bottom of polypropylene tubes. The resulting clots were referred to as having high (H), intermediate (I), and low (L) thrombin/fibrinogen ratios.

#### Quantification of purified FXIIa activity by chromogenic assays

The fibrin clots were immediately incubated with 20 μL of the specific thrombin inhibitor, lepirudin (Schering AG, Berlin, Germany, 2.5 mg/mL) for 10 minutes at 37°C. Thereafter, 1 μg of purified FXII (Enzyme Research Laboratories, Swansea, UK) in 100 μL Tris buffer (50 mM Tris, 175 mM NaCl, 7.5 mM EDTA, pH 8.4) was added to the fibrin clots and incubated for an additional 10 minutes at 37°C: 100 μL of S-2302 (2.5 mM) and S-2238 (1 mM) (both from Haemochrom Diagnostica) was added separately and incubated for 5 minutes at 37°C. The reaction was stopped by adding 100 μL of 20% citric acid, and the absorbance at 405 nm was measured in the supernatant after centrifugation at 2200 × *g* for 2 minutes.

**Figure 1:**
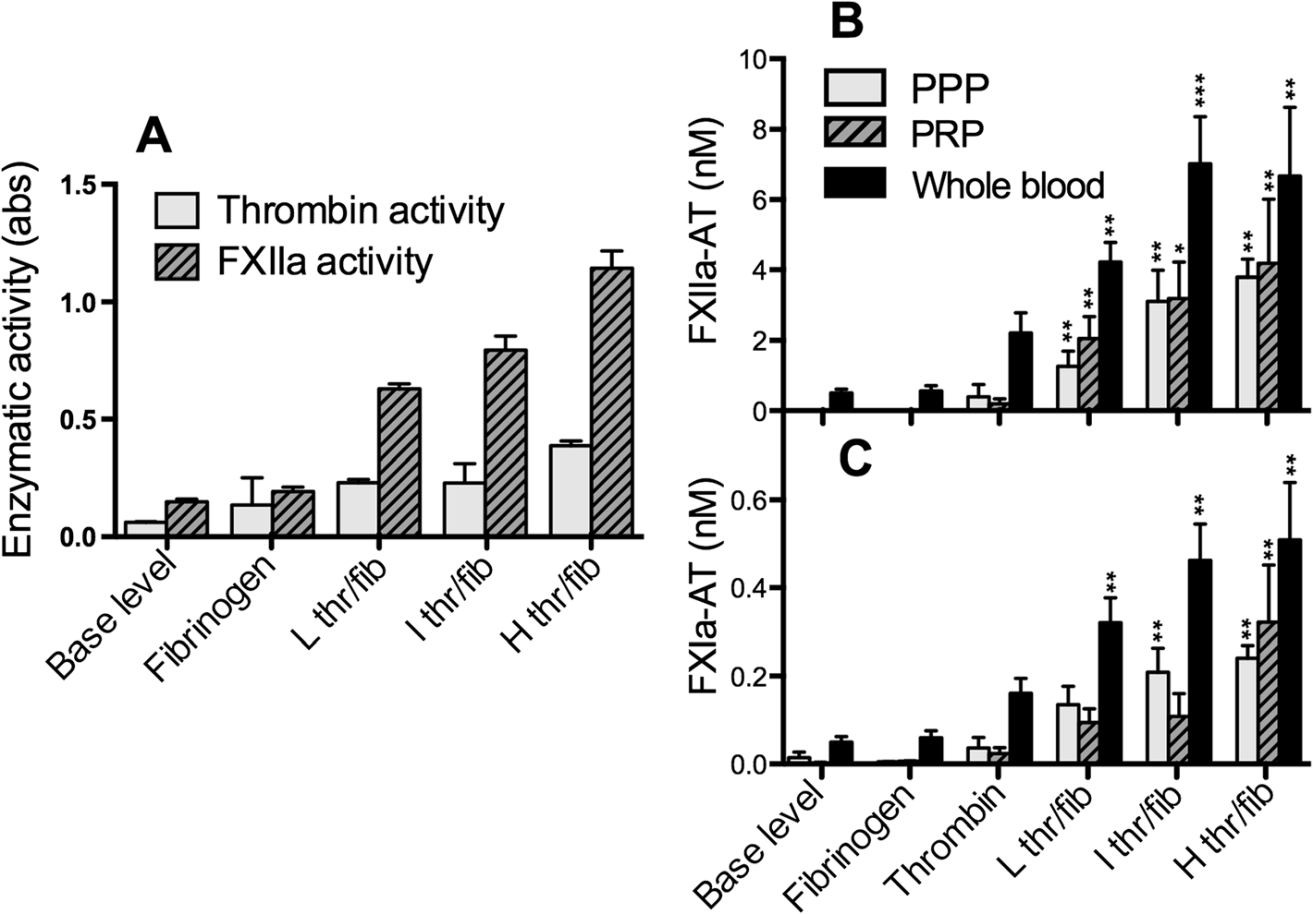
**Fibrin activates FXII and elicits FXIIa-antithrombin (AT) complex formation in blood.** Fibrin clots were produced at the bottom of tubes. Three different thrombin (thr)/ fibrinogen (fib) ratios generated clots with low (L), intermediate (I), and high (H) density. **(A)** The clots or native fibrinogen were incubated with 1 μg of purified FXII, and the enzymatic activity of FXIIa and thrombin was measured using the chromogenic substrates S-2302 (FXIIa) and S-2238 (thrombin). Data are presented as the means ± standard error of the mean (SEM) (n = 3). **(B**-**C)** The clots, native fibrinogen, and thrombin were incubated with lepirudin-anticoagulated platelet-poor plasma (PPP), platelet-rich plasma (PRP), and whole blood. The generation of FXIIa-AT **(A)** and FXIa-AT **(B)** by the clots in all blood systems was measured by ELISA. Data are presented as the mean ± SEM (n = 5), and differences compared with the base level are shown (Kruskall-Wallis test with Dunn’s multiple comparison).

S-2302 was used to quantify FXIIa, and S-2238 as a control to quantify thrombin since thrombin also can cleave S-2302. The manufacturer reports substrate turnover values (expressed as ΔA/min with protease concentration 4*10^-9^ M) of 0.480 when S-2238 is used to detect thrombin activity, and of 0.190 when S-2302 is used to detect kallikrein activity. Furthermore, the specificity of FXIIa for S-2302 is substantially lower than that for kallikrein [8]. Consequently, data (absorbance) presented in Figure 1A overestimate the generation of thrombin compared to FXIIa activity.

#### Quantification of FXIIa-serpin complexes in whole blood

Lepirudin-anticoagulated whole blood (700 μL), PRP (350 μL), or PPP (350 μL) from five different donors were added to the fibrin clots. The PPP samples were incubated for 30 minutes, and the others were incubated for 15 minutes, all at 37°C. The experiments with whole lepuridin-anticoagulated blood were also repeated in the presence of 5 μM (final blood concentration) of the low molecular weight thrombin inhibitor Melagatran (Astra Zeneca, Gothenburg, Sweden), which was added to the fibrin clots 5 minutes before the addition of the blood.

### Activation of FXII induced by glass

Citrate anti-coagulated PPP (350 μL) from six different donors was incubated in glass tubes (borosilicate glass 10 × 75 mm, Kimble-Kontes, Vineland, NJ, USA), with and without the addition of calcium chloride (CaCl_2_) at a final concentration of 10 mM, for 30 minutes at 37°C. The reaction was blocked by adding EDTA to the samples to give a final plasma concentration of 10 mM. All samples were centrifuged at 2200 × *g* for 15 minutes at 4°C, and the plasma obtained was stored at -70°C until analyzed for FXII- and FXI-serpin complexes.

### Electrophoresis and western blotting

Fibrinogen (Haemochrom Diagnostica) or increasing concentrations (0.025 to 33.0 μg/mL) of thrombin (Hoffmann-La Roche) were incubated with purified human FXII (Enzyme Research Laboratories, 2.5 μg/mL) for 15 minutes at 37°C. The proteins were analyzed by SDS-PAGE (10% gels) under reducing conditions followed by either staining with Coomassie brilliant blue or western blotting as previously described [22]. The polyvinylidene fluoride (PVDF) membranes were incubated with sheep anti-human FXII (the binding site) diluted 1:250. The membranes were then incubated with horseradish peroxidase (HRP)-conjugated anti-sheep Ig (Dako, Glostrup, Denmark) diluted 1:500 and developed with diaminobenzidine (Sigma-Aldrich, St Louis, MO, USA).

### Statistical analyses

Non-parametric statistical analyses as indicated in the figure legends were performed using the commercial program GraphPad Prism Version 4 (GraphPad Software, Inc., San Diego, CA, USA). Data in the text section are presented as medians and interquartile ranges. *P*-values <0.05 were considered significant and in the figures are presented as ^*^ = *P* <0.05, ^**^ = *P* <0.01 or ^***^ = *P* <0.0001.

## Results

### Fibrin activates FXII into FXIIa

Fibrin clots were produced at the bottom of plastic tubes, and these pre-made clots were incubated with purified FXII or with blood containing lepirudin. In the purified system, we observed significant FXII activation induced by the fibrin network as assessed by the chromogenic substrate S-2302 (Figure 1A). As the sensitivity and specificity of substrate S-2238 for thrombin is considerably higher than that of S-2302 for FXIIa, the low enzymatic thrombin activity detected with the chromogenic substrate S-2238 is overestimated, and confirmed that the cleavage of S-2302 originated from activated FXII.

The fibrin clots caused a significant generation of FXIIa-AT and FXIa-AT in PPP, PRP, and whole blood, with the highest levels in whole blood (Figure 1B-C), whereas the corresponding C1INH complexes were not detectable (not shown). The generation of AT complexes demonstrated that activation of FXII occurred in the blood/plasma systems and that AT was the preferred regulator. It also indicated that the activation proceeded to FXI, as thrombin in the presence of lepirudin could not contribute to FXI activation. Fibrinogen alone did not induce AT complexes, and purified thrombin alone triggered only a minor generation in whole blood.

To further certify that any remaining thrombin did not lead to FXII activation, lepirudin inhibition was complemented with the low molecular weight thrombin-inhibitor Melagatran, which is able to better penetrate the fibrin clots. Still, no difference in FXIIa generation was observed (data not shown). We also incubated purified human FXII in the presence of increasing concentrations of thrombin without observing any cleavage of FXII by SDS-PAGE and/or western blot analysis (data not shown).

### Fibrin promotes AT inhibition of activated FXII

**Figure 2:**
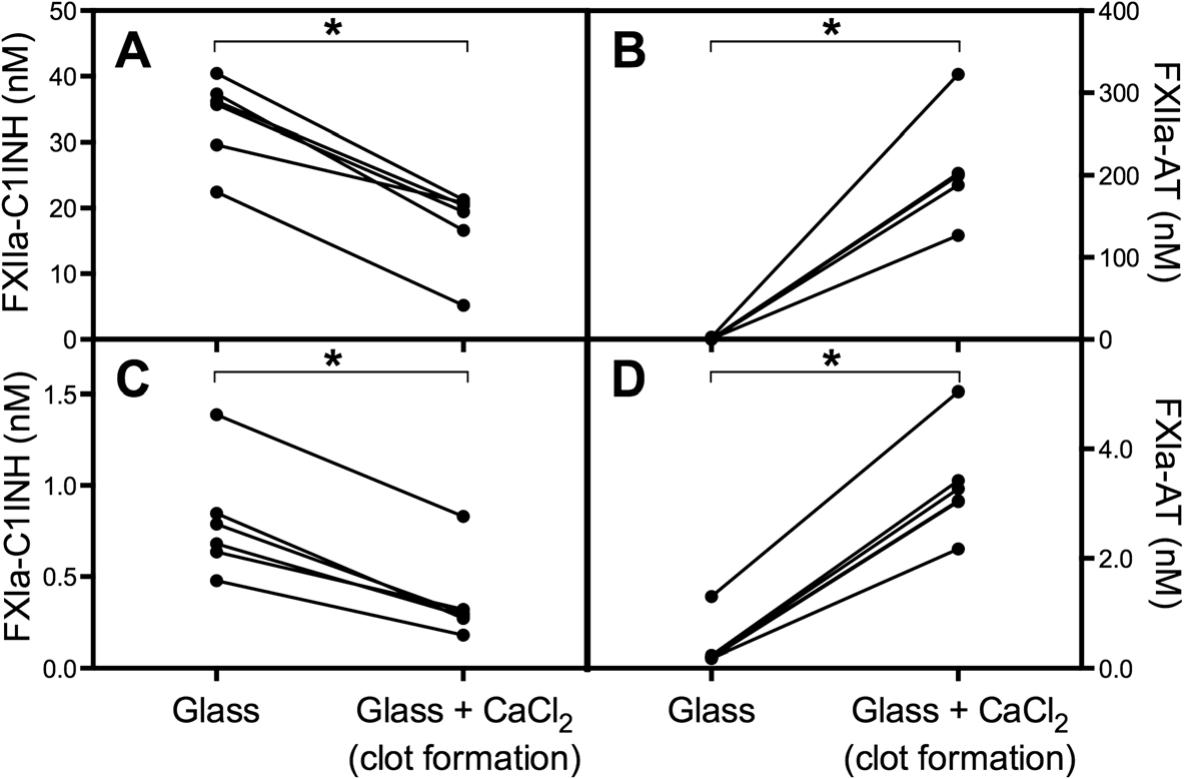
**A reciprocal relationship exists between the generation of FXIIa-antithrombin (AT) and FXIIa-C1 inhibitor (C1INH) in clotting plasma.** Citrated plasma from healthy donors, with and without the addition of calcium, was incubated in glass tubes, which elicits FXII activation on the glass surface. The levels of FXIIa-C1INH **(A)**, FXIIa-AT **(B)**, FXIa-C1INH **(C)**, and FXIa-AT **(D)** were analyzed by sandwich ELISA. Each line connects data from one individual (n = 6), and statistical significance was determined using Wilcoxon’s signed-rank test.

Based on our initial experiment demonstrating that fibrin clots activated FXII, we wanted to corroborate that fibrin could also enhance the affinity or accessibility of AT for FXIIa. Artificial material-induced FXII activation, which is regulated by C1INH [17] was triggered in citrated plasma by the surface in glass tubes. Both FXIIa-C1INH and FXIa-C1INH were generated without any generation of the corresponding AT complexes. However, when the plasma was allowed to coagulate and form fibrin clots, triggered by re-calcification, the levels of the C1INH-complexes decreased and, reciprocally, FXIIa-AT and FXIa-AT were generated. This reflects an increased tendency of AT to inhibit FXIIa and FXIa and a corresponding decline in the C1INH complexes when blood clots and fibrin are present (Figure 2).

### Levels of FXIIa-serpin complexes in SLE patients and healthy controls

**Figure 3:**
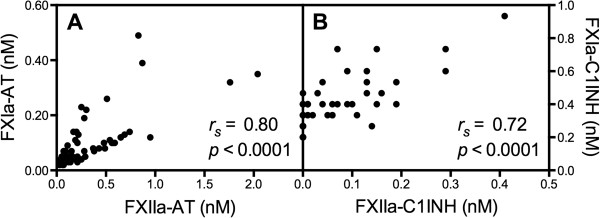
**Antithrombin (AT) and C1 inhibitor (C1INH) complexes, respectively, correlated with each other in systemic lupus erythematosus (SLE) patients.** Complexes formed between activated FXII and FXI and the serpins AT and C1INH were measured by sandwich ELISA in plasma samples from SLE patients (n = 69). FXIIa-AT correlated with FXIa-AT **(A)**, whereas FXIIa-C1INH correlated with FXIa-C1INH **(B)**. Spearman’s *r* (*r*_*s*_) coefficients and *P*-values are given for each calculation.

Because patients with SLE exhibit persistent platelet activation and an increased risk of thrombotic events, we assessed FXIIa-serpin complexes in an SLE patient group enriched in patients with a history of VD. Plasma samples were analyzed for the presence of complexes formed between FXIIa and FXIa with the serpins AT and C1INH. Patients with SLE exhibited an altered formation of FXIIa complexes. In particular, we observed decreased FXIIa-C1INH levels in the SLE patients when compared with baseline levels in control individuals (0.00 (0.00 to 0.07) versus 0.08 (0.03 to 0.13) nM), *P* <0.0001). In contrast, the levels of FXIIa-AT tended to increase in the SLE patients (Additional file 3: Table S2), and there was a negative correlation between FXIIa-C1INH and FXIIa-AT (*r*_s_ = -0.24, *P* = 0.0416). The levels of FXIa-AT were strongly correlated with FXIIa-AT, and correspondingly, FXIa-C1INH was strongly correlated with FXIIa-C1INH (Figure 3A-B).

### Altered levels of FXIIa-serpin complexes are associated with previous thrombotic events in SLE patients

**Figure 4:**
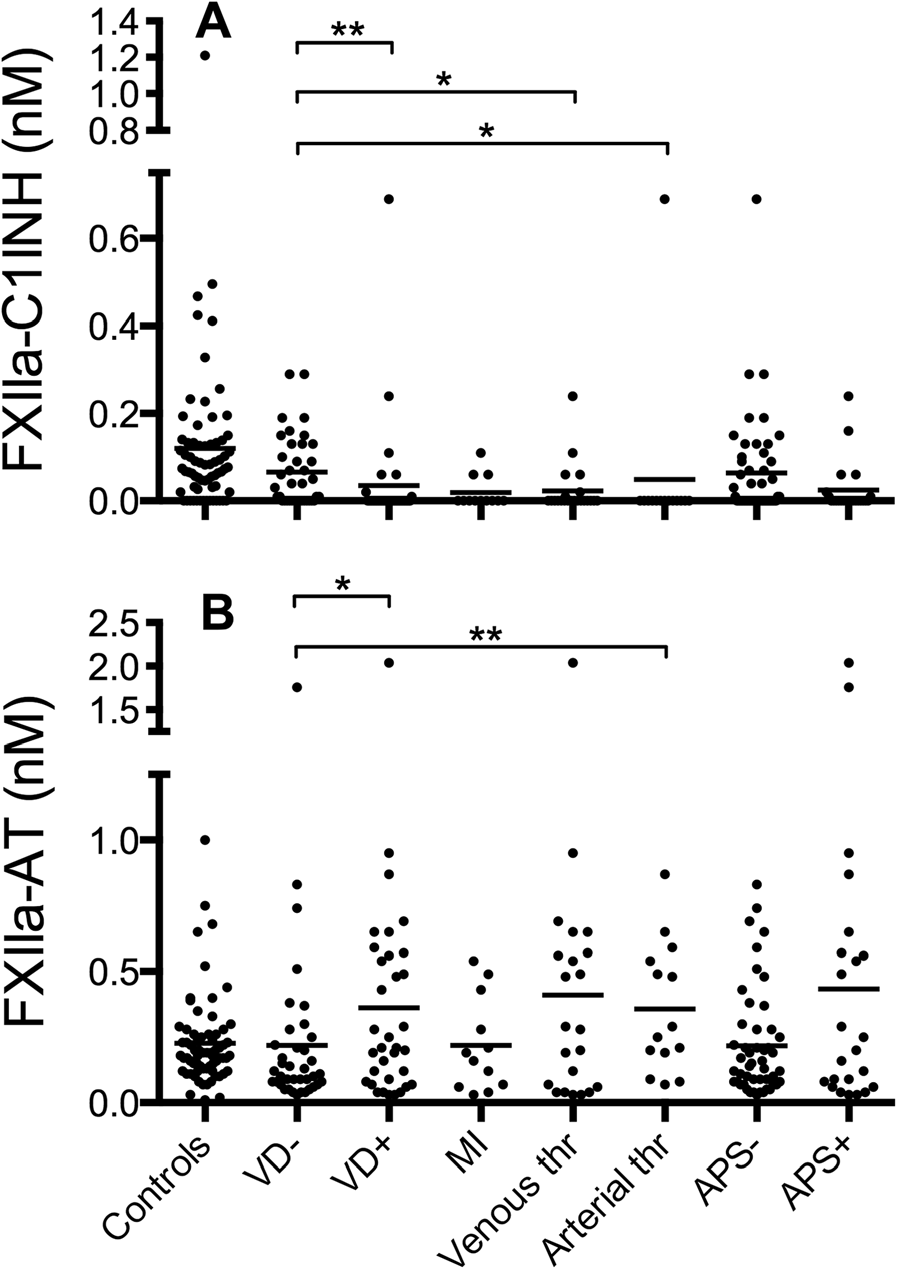
**Altered levels of FXIIa-serpin complexes in systemic lupus erythematosus (SLE) patients with previous vascular disease (VD).** The plasma levels of FXIIa-C1INH **(A)** and FXIIa-AT **(B)** were assessed by sandwich ELISA in plasma from SLE patients (n = 69) and healthy controls (n = 68). Patients without previous vascular disease (VD-) were compared with those with previous VD (VD+). The VD + group was further divided into three subgroups: myocardial infarction (MI), venous thrombosis (Venous thr), and arterial thrombosis (Arterial thr). Furthermore, patients with antiphospholipid antibody syndrome (APS+) were compared with those without (APS-). In several samples, the levels of FXIIa-C1INH were below the level of detection (0.01 nM), and these data points are not visible in the figure. The numbers of individuals in the various groups with such data are as follows: controls 11/68, VD- 14/38, VD + 24/31, MI 7/10, Venous thr 14/20, Arterial thr 12/13, APS- 25/49, and APS + 13/20. The lines represent the mean values, and statistical significance was determined with the non-parametric Kruskall-Wallis test with Dunn’s multiple comparison (differences when compared with the control are not shown).

To further investigate whether the alterations in FXIIa-serpin complexes could be involved in the pathogenic mechanisms leading to thrombotic events in SLE, we categorized the patients into those with and those without a history of VD. The VD group was further divided into three subgroups: myocardial infarction (MI), venous thrombosis (pulmonary embolism or deep venous thrombosis), and arterial thrombosis (12/13 with cerebrovascular incidents) (Additional file 2: Table S1). We observed distinct differences in the plasma levels of both FXIIa-AT and FXIIa-C1INH. Increased levels of FXIIa-AT and the lowest levels (in most cases equivalent to no detected complexes at all) of FXIIa-C1INH were found in patients with previous VD (Figure 4). When we compared the three subgroups of VD, we observed this reciprocal pattern in those patients with previous venous or arterial thrombosis but not in those with previous MI, although the difference for FXIIa-AT was only statistically significant in the case of the arterial thrombosis subgroup.

We also compared patients with a history of anti-phospholipid antibody syndrome (APS) to those without APS [23]. Patients with APS exhibited a tendency toward lower levels of FXIIa-C1INH and higher levels of FXIIa-AT than did those without APS (not significant).

### The generation of FXIIa-AT is strongly linked with activated platelets

**Figure 5:**
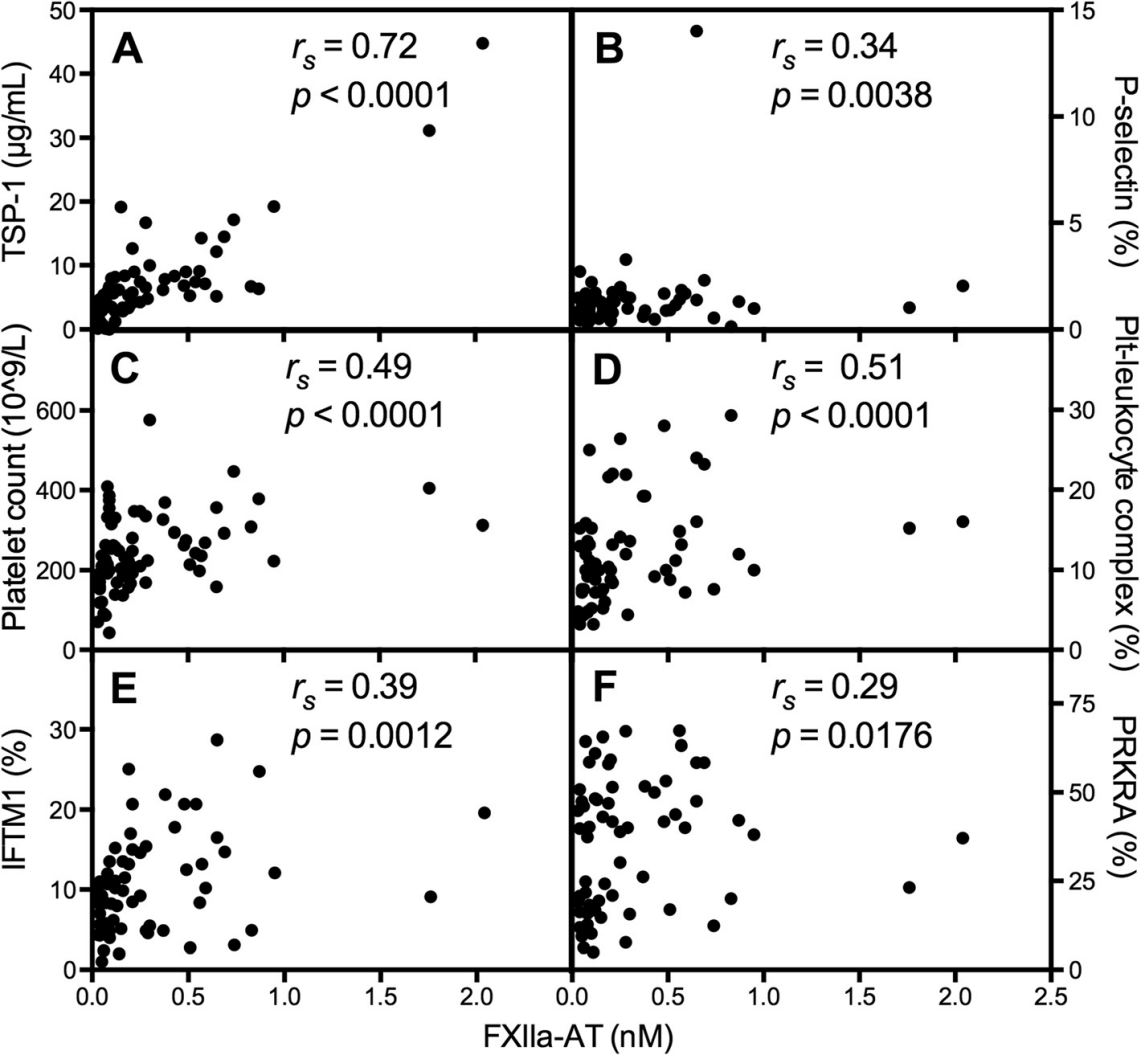
**The occurrence of FXIIa-antithrombin (AT) is clearly linked to platelet activation.** Plasma levels of thrombospondin 1 (TSP-1) **(A)**, platelet expression of P-selectin (CD62P) **(B)**, platelet count **(C)**, platelet-leukocyte complexes **(D)**, platelet intracellular levels of the type I IFN-regulated proteins (IFITM1) and protein kinase IFN-inducible double-stranded RNA dependent activator (PRKRA) **(E and F)**, all parameters reflecting platelet activation, were assessed in systemic lupus erythematosus (SLE) patients (n = 69). All parameters were shown to statistically correlate with FXIIa-AT tested with Spearman’s rank-order correlation coefficients (*r*_*s*_).

The levels of FXIIa-AT were closely correlated with those of the two platelet markers: thrombospondin (TSP)-1 in plasma and platelet P-selectin exposure (Figure 5A-B). The platelet counts and levels of platelet-leukocyte complexes were also correlated with those of FXIIa-AT (Figure 5C-D). None of the above-mentioned markers allowed us to distinguish between patients with and without previous VD. The levels of IFITM1 and PRKRA, which have recently been reported to be up-regulated in SLE patients with VD and in those associated with activated platelets [17], were positively correlated with those of FXIIa-AT (Figure 5E-F). The levels of FXIa-AT were also correlated with all platelet activation markers except PRKRA (data not shown). The levels of FXIa-C1INH or FXIa-C1INH were not correlated with any of the platelet activation markers except for the intracellular levels of PRKRA and IFITM1, which were negatively correlated with FXIIa-C1INH (Additional file 4: Table S3).

The plasma levels of C1q, C3, and C4, which are an indirect and insensitive mirror of complement activation and immune complex disease, were not correlated to any of the FXIIa-AT or FXIIa-C1INH complexes. No association was found between the FXIIa-serpin complexes and the systemic lupus erythematosus disease activity index (SLEDAI) at the time the samples were drawn.

Except for patients treated with warfarin, which tended to exhibit the highest levels of FXIIa-AT, we observed no association between any of the AT and C1INH complexes and any of the other most frequently used treatments or with other disease manifestations, such as glomerulonephritis or arthritis (data not shown). It should be noted that all of the warfarin-treated patients exhibited previous thrombosis except for six patients who all had very low levels of FXIIa-AT, which reflects that warfarin lowers the risk of thrombosis, and thereby decreases the FXIIa-AT levels.

### The alterations in the levels of FXIIa-serpin complexes points to new biomarkers for VD risk evaluation in SLE patients

The odds ratios (OR) for VD with respect to the two types of FXIIa-serpin complexes (Table 1) were calculated. Based on receiver operating characteristic (ROC) curve analyses and the Youden index, the cutoff value for FXIIa-C1INH was set to <0.01 nM, which corresponded to the detection limit of the assay, and for FXIIa-AT to >0.40 nM, which corresponded to the 90% percentile of the controls. The OR for VD was 8.9 if the levels of FXIIa-C1INH were low and 6.1 if the levels of FXIIa-AT were high. If the complexes were combined and both the criteria were fulfilled, the OR increased to 23.4. When the OR was calculated in this material with respect to the occurrence of anti-cardiolipin antibodies, the OR was 2.0, but there was no significant difference in the VD outcome.

**Table 1:** **FXIIa**-**serpin complexes and aCL antibodies in association with VD in SLE patients**

	**VD-, n (%)**	**VD+, n (%)**	***P-value***^**a**^	**OR (95% CI)**
**All**	**38 (55)**	**31 (45)**		
FXIIa-C1INH <0.01 nM^b^	14 (37)	24 (77)	*P* <0.0001	8.9 (2.8, 28.5)
FXIIa-AT >0.40 nM^c^	4 (11)	13 (42)	*P* = 0.0043	6.1 (1.7, 21.6)
Both low FXIIa-C1INH	1 (3)	12 (39)	*P* <0.0001	23.4 (2.8, 194)
and high FXIIa-AT				
aCL antibodies	12 (32)	15 (48)	n.s.	2.0 (0.8, 5.4)

SLE, systemic lupus erythematosus; VD, vascular disease; F, factor; C1INH, C1 inhibitor; AT, antithrombin; Acl, anti-cardiolipin; OR, odds ratio; n.s., not significant. ^a^Fisher's exact test; ^b^0.01 nM = detection limit of the assay; ^c^0.40 nM = 90% percentile of controls.

## Discussion

We have previously shown that activation of platelets induces activation of FXII that is regulated by AT, which is reflected by the formation of large amounts of FXIIa-AT and FXIa-AT complexes in clotted blood [8,17]. In the present work, we demonstrate that fibrin formation is an essential mechanism that drives the activation of FXII and the subsequent formation of FXIIa-AT complexes. We also found that an SLE patient group enriched in patients with previous VD exhibited altered levels of FXIIa-complexes when compared with healthy controls and that increased levels of FXIIa-AT and decreased levels of FXIIa-C1INH were associated with VD and previous thrombotic events. These results are in line with recent data indicating an important role of FXII in thrombus formation.

In the present study, we asked whether fibrin clots could trigger FXII activation and if so, how it was regulated. Preformed fibrin clots were shown to activate purified FXII, and when incubated in blood, there was a substantial generation of both FXIIa-AT and FXIa-AT complexes, similar to what we previously have shown to occur in clotting blood. Thrombin cannot activate FXII, and with the simultaneous use of the antithrombin compounds melagatran and lepirudin, we verified that activation of FXII was not mediated by thrombin. These findings are consistent with a recent study demonstrating that fibrin binds FXII and α-FXIIa [24]. Complementing these findings, our data obtained using chromogenic substrates and measuring FXIIa-AT complexes, demonstrate that FXII is specifically activated by the preformed fibrin clots.

We also observed a reciprocal relationship between the generation of FXIIa-AT and FXIIa-C1INH complexes in plasma if fibrin generation and clotting were triggered *in vitro*. Fibrin formation promoted the generation of AT complexes and seemed to increase the affinity, or the accessibility, of AT for FXIIa and FXIa, competing out C1INH and leading to the generation of FXIIa-AT and FXIa-AT complexes. Thus, fibrin seems to act as a physiological heparin-like co-factor.

In previous studies, we and others, have shown that platelet activation and an increased generation of fibrin continuously occurs *in vivo* in SLE, particularly in those patients with a history of VD [9,22,25,26]. Given the strong association between FXIIa-AT levels in plasma and platelet activation *in vitro*[8,17], we expected to find increased levels of these complexes in SLE patients, with the highest levels in those with VD. When SLE patients with and without a history of VD were compared, we found significantly increased levels of FXIIa-AT in those who had VD. Higher levels of FXIIa-AT were found particularly in those patients with previous venous and arterial thrombosis. With the exception of the previously reported IFITM1 and PRKRA in platelets [20], none of the other markers of platelet activation that we examined allowed us to differentiate between the patients with previous VD and those without. A history of previous anti-cardiolipin antibodies was also uninformative.

Similar to the situation in the *in vitro* experiments, the high FXIIa-AT levels *in vivo* were reciprocal to those of FXIIa-C1INH, which were decreased in the SLE patients and particularly in those with a history of thrombotic events, where in many cases no FXIIa-C1INH could be detected. The decrease in FXIIa-C1INH was corroborated by a report of a study that investigated patients with coronary heart disease and stroke [27]. In that study, patients with VD exhibited significantly lower levels of FXIIa-C1INH complexes, particularly in the case of smokers, than did controls. Based on our *in vitro* experiments where fibrin gave rise to a switch from FXIIa-C1INH to FXIIa-AT complexes (see above), the generation of soluble fibrin and microthrombi, which preferentially promote AT regulation of FXIIa, is a possible cause of the decreased FXIIa-C1INH levels.

Further support for a link between platelet-induced fibrin generation and the generation of AT complexes was that both FXII-AT and FXIa-AT but none of the C1INH complexes were correlated with several platelet parameters, for example, TSP-1, P-selectin exposure, platelet count, and platelet-leukocyte complexes. Moreover, FXIIa-AT levels were correlated with the two type I IFN-regulated proteins, IFITM1 and PRKRA, detected in platelets. The up-regulation of these two proteins in platelets has previously been shown to be associated with platelet activation and VD in SLE [20].

The levels of FXIIa-AT were strongly correlated with those of FXIa-AT in the SLE patients. This was also the case for the corresponding C1INH complexes. These results suggest that the regulation of the protease with AT or C1INH occurred under different circumstances. The results may indicate that the initial activation of FXII occurred at different sites, where clot formation may represent the milieu where AT complexes are formed. Similar to our *in vitro* experiments, we also observed a negative correlation between FXIIa-AT and FXIIa-C1INH complexes in the SLE patients. This further emphasizes different mechanisms of FXII activation and complex formation, and the likelihood that soluble fibrin or microthrombi may be the explanation for the altered ratios of FXIIa complexes. Furthermore, these *in vivo* data corroborate that fibrin possesses heparin-like AT co-factor activity.

Today, assessment of anti-cardiolipin antibodies and lupus anticoagulants is the state-of-the-art approach to evaluate the risk of thrombotic events in SLE patients [23]. Anti-phospholipid antibodies are linked to, but not strictly correlated with VD and thrombotic events, as is also reflected in the present but limited study. By itself, our analytic approach is not sufficiently specific and needs to be complemented with other analyses to produce an accurate assessment of the risk of future thrombotic events. FXIIa-serpin complexes, which were strongly associated with VD, may be emerging analyses for the future evaluation of the risk of thrombotic events in SLE patients. However, these analyses need to be validated in prospective studies before they can be used alone or in combination as a means for gauging the risk of thrombotic events in SLE.

## Conclusion

This study shows that activation of the contact system is involved in the pathophysiology of SLE and that the activation of the contact system is likely to be triggered by fibrin. We show that the formation of FXIIa-AT complexes is associated with thrombotic events and that generation of these complexes represents a promising potential biomarker for evaluation of the risk of thrombotic events in SLE.

## Abbreviations

APSP: Antiphospholipid antibody syndrome; AT: Antithrombin; C1INH: C1 inhibitor; EDTA: Ethylenediaminetetraacetic acid; ELISA: Enzyme-linked immunosorbent assay: F, Factor; HK: High molecular-weight kininogen; IC: Immune complex; IFITM1: Type I interferon-induced transmembrane protein 1; IFN: Type I interferon; MI: Myocardial infarction; OR: Odds ratio; PPP: Platelet-poor plasma; PRKRA: Protein kinase IFN-inducible double-stranded RNA dependent activator; PRP: Platelet-rich plasma; SLE: Systemic lupus erythematosus; SLEDAI: Systemic lupus erythematosus disease activity index; TAT: Thrombin-antithrombin; TSP: Thrombospondin; VD: Vascular disease.

## Competing interests

The authors declare that they have no competing interest.

## Authors’ contributions

JB, KNE and BN have designed and performed the preclinical experiments. They have also analyzed and interpreted all the data. JB has performed the statistical analyses. JB, CL, and AB have acquired the clinical data. JB has drafted the manuscript, while BN, KNE, CL, AB have edited the manuscript. All authors have read and approved the final manuscript.

## Supplementary Material

Additional file 1: Figure S1 Schematic illustration of contact activation on a surface. The contact activation process is initiated through autoactivation of factor XII (black arrow). Activated factor XII activates factor XI, initiating the intrinsic pathway of the coagulation cascade (red arrows), and prekallikrein, initiating the kallikrein-kinin system (green arrows) that releases the pro-inflammatory and vasoactive peptide bradykinin. Factor XII activation is accelerated by feedback activation of kallikrein. High molecular-weight kininogen functions as a co-factor for kallikrein and factor XI by facilitating their binding to surfaces. The serpins C1 inhibitor and antithrombin are able to inhibit the activated proteases of the contact system by forming irreversible complexes with them. Contact activation initiated by biomaterial surfaces, and presumably also endothelial cells, is regulated by C1 inhibitor, whereas activation induced by activated platelets and occurring in clot formation is regulated by antithrombin. F, factor; a, activated; HK, high molecular-weight kininogen; PK, prekallikrein; KK, kallikrein; BK, bradykinin; C1INH, C1 inhibitor; AT, antithrombin. Click here for file

Additional file 2: Table S1 Clinical characteristics of the systemic lupus erythematosus (SLE) patients according to American College of Rheumatology (ACR) criteria and occurrence of cardiovascular events at any time during disease. Click here for file

Additional file 3: Table S2 Levels of FXII and protease-serpin complexes in healthy controls and systemic lupus erythematosus (SLE) patients. Click here for file

Additional file 4: Table S3 Correlations of FXIIa- C1 inhibitor (C1INH), FXIIa-antithrombin (AT), and thrombin-antithrombin (TAT) levels with contact system protease-serpin complexes and with markers related to platelet activation and clotting in systemic lupus erythematosus (SLE) patients. Click here for file
