# Supplementary material for: Contact activation products are new potential biomarkers to evaluate the risk of thrombotic events in systemic lupus erythematosus
Source: Arthritis Res Ther. 2013 Dec 4;15(6):R206. doi: 10.1186/ar4399 (PMC3979000; doi:10.1186/ar4399)
Supplement: Additional file 2: Table S1 — Clinical characteristics of the systemic lupus erythematosus (SLE) patients according to American College of Rheumatology (ACR) criteria and occurrence of cardiovascular events at any time during disease. [file ar4399-S2.docx]

| **Table S1. Clinical characteristics of the SLE patients according to** | |
| --- | --- |
| **ACR criteria and occurrence of cardiovascular events at any** | |
| **time during disease** | |
|  | n = 69 |
| Malar rash, % | 61 |
| Discoid rash, % | 30 |
| Photosensitivity, % | 64 |
| Oral ulcers, % | 30 |
| Arthritis, % | 81 |
| Serositis, % | 65 |
| Renal disease, % | 42 |
| Neurologic disorder, % | 9 |
| Hematologic manifestations, % | 52 |
| Leukopenia, % | 39 |
| Lymphopenia, % | 28 |
| Thrombocytopenia, % | 20 |
| Immunology, % | 67 |
| ANA, % | 100 |
| Anti-DNA antibodies, % | 71 |
| Anti-cardiolipin antibodies, % | 45 |
| Anti-phospholipid antibody syndrome, % | 29 |
| Vascular disease, % | 45 |
| Median time since event, year (range) | 8 (1-36) |
| Venous thrombosis, % | 29 |
| Median time since event, year (range) | 13 (3-36) |
| Arterial thrombosis, % | 19 |
| Median time since event, year (range) | 8 (1-18) |
| Myocardial infarction, % | 15 |
| Median time since event, year (range) | 6 (2-30) |
| SLE = systemic lupus erythematosus, | |
| ACR = American College of Rheumatology | |
