# Supplementary material for: Contact activation products are new potential biomarkers to evaluate the risk of thrombotic events in systemic lupus erythematosus
Source: Arthritis Res Ther. 2013 Dec 4;15(6):R206. doi: 10.1186/ar4399 (PMC3979000; doi:10.1186/ar4399)
Supplement: Additional file 3: Table S2 — Levels of FXII and protease-serpin complexes in healthy controls and systemic lupus erythematosus (SLE) patients. [file ar4399-S3.docx]

| **Table S2. Levels of FXII and protease-serpin complexes in healthy controls and SLE patients** | | | |
| --- | --- | --- | --- |
|  | Healthy controls (n=68) | SLE patients (n=69) | *p*-value * |
| TAT, µg/L | 2.0 (0.3, 1.2, 2.4, 14.9) ** | 2.4 (0.7, 1.3, 4.6, 50.8) | 0.0182 |
| FXIIa-C1INH, nM | 0.08 (0.00, 0.03, 0.13, 1.21) | 0.00 (0.00, 0.00, 0.07, 0.41) | <0.0001 |
| FXIIa-AT, nM | 0.18 (0.01, 0.12, 0.26, 1.00) | 0.16 (0.03, 0.07, 0.38, 2.04) | n.s. |
| FXIa-C1INH, nM | 0.05 (0.04, 0.05, 0.06, 0.24) | 0.06 (0.03, 0.05, 0.07, 0.14) | <0.0001 |
| FXIa-AT, nM | 0.11 (0.03, 0.09, 0.17, 0.38) | 0.07 (0.02, 0.04, 0.16, 0.49) | 0.0005 |
| SLE = systemic lupus erythematosus, T = thrombin, AT = antithrombin, F = factor, C1INH = C1 inhibitor, | | | |
| n.s. = not significant, * Mann Whitney test, ** Median (minimum, 25% percentile, 75% percentile, maximum) | | | |
