# Supplementary material for: Contact activation products are new potential biomarkers to evaluate the risk of thrombotic events in systemic lupus erythematosus
Source: Arthritis Res Ther. 2013 Dec 4;15(6):R206. doi: 10.1186/ar4399 (PMC3979000; doi:10.1186/ar4399)
Supplement: Additional file 4: Table S3 — Correlations of FXIIa- C1 inhibitor (C1INH), FXIIa-antithrombin (AT), and thrombin-antithrombin (TAT) levels with contact system protease-serpin complexes and with markers related to platelet activation and clotting in systemic lupus erythematosus (SLE) patients. [file ar4399-S4.docx]

| **Table S3. Correlations of FXIIa-C1INH, FXIIa-AT, and TAT levels with contact system protease-serpin complexes and with markers related to platelet activation and clotting in SLE patients** | | | | | | |
| --- | --- | --- | --- | --- | --- | --- |
|  | FXIIa-C1INH | p-value * | FXIIa-AT | p-value | TAT | *p*-value |
| FXIIa-C1INH | - | - | -0.24 ** | 0.0416 | 0.28 | 0.0159 |
| FXIIa-AT | -0.24 | 0.0416 | - | - | 0.36 | 0.0020 |
| FXIa-C1INH | 0.72 | <0.0001 | - | n.s. | - | n.s. |
| FXIa-AT | - | n.s. | 0.80 | <0.0001 | 0.26 | 0.0280 |
| TAT | 0.28 | 0.0159 | 0.36 | 0.0020 | - | - |
| Platelet count | - | n.s. | 0.49 | <0.0001 | - | n.s. |
| Thrombospondin-1 | - | n.s. | 0.72 | <0.0001 | - | n.s. |
| P-selectin | - | n.s. | 0.34 | 0.0038 | - | n.s. |
| PRKRA | -0.36 | 0.0029 | 0.29 | 0.0176 | 0.27 | 0.0300 |
| IFITM1 | -0.30 | 0.0146 | 0.39 | 0.0012 | - | n.s. |
| Platelet-leukocyte complex | - | n.s. | 0.51 | <0.0001 | - | n.s. |
| SLE = systemic lupus erythematosus, T = thrombin, AT = antithrombin, F = factor, C1INH = C1 inhibitor, | | | | | | |
| PRKRA = protein kinase, interferon-inducible double-stranded RNA dependent activator, | | | | | | |
| IFITM1 = interferon-induced transmembrane protein 1, n.s. = not significant. | | | | | | |
| * Spearman correlation, **Spearman r | | | | | | |
