# Supplementary material for: Contact activation products are new potential biomarkers to evaluate the risk of thrombotic events in systemic lupus erythematosus
Source: Arthritis Res Ther. 2013 Dec 4;15(6):R206. doi: 10.1186/ar4399 (PMC3979000; doi:10.1186/ar4399)
Supplement: Supplementary file 4 [file ar4399-S1.docx]

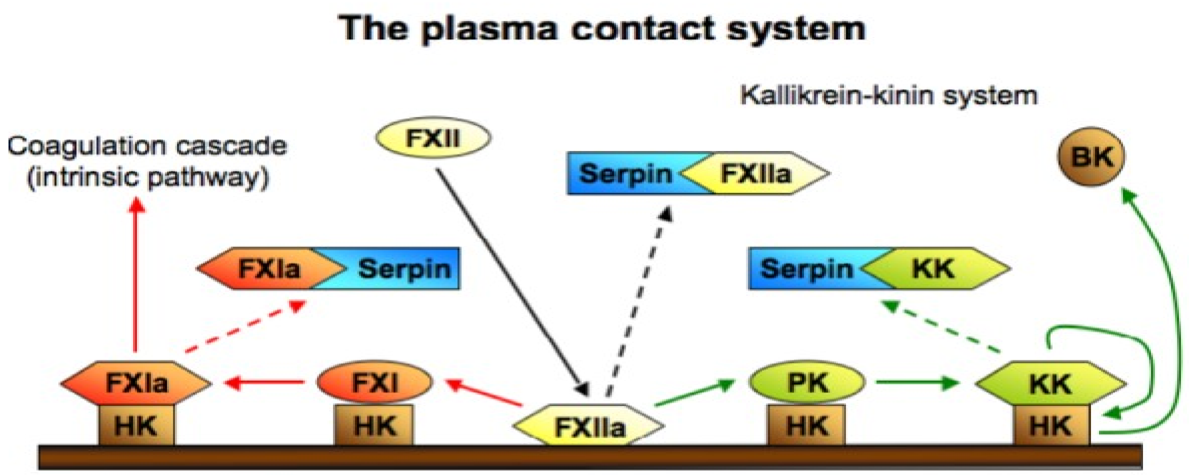
The plasma contact system
Kallikrein-kinin system
Coagulation cascade
(intrinsic pathway)
FXII
Serpin
FXIIa
BK
FXIa
Serpin
Serpin
KK
FXIa
FXI
PK
KK
HK
HK
FXIIa
HK
HK
